# Frameworks for measuring population health: A scoping review

**DOI:** 10.1371/journal.pone.0278434

**Published:** 2024-02-13

**Authors:** Sze Ling Chan, Clement Zhong Hao Ho, Nang Ei Ei Khaing, Ezra Ho, Candelyn Pong, Jia Sheng Guan, Calida Chua, Zongbin Li, Trudi Lim, Sean Shao Wei Lam, Lian Leng Low, Choon How How

**Affiliations:** 1 Health Services Research Centre, SingHealth, Singapore, Singapore; 2 Health Services and Systems Research, Duke-NUS Medical School, Singapore, Singapore; 3 Centre for Population Health Research and Implementation, SingHealth Regional Health System, Singapore, Singapore; 4 Health Services Research, Changi General Hospital, Singapore, Singapore; 5 School of Biological Sciences, Nanyang Technological University, Singapore, Singapore; 6 Care and Health Integration, Changi General Hospital, Singapore, Singapore; 7 Preventive Medicine Residency, National University Health System, Singapore, Singapore; 8 School of Computing and Information Systems, Singapore Management University, Singapore, Singapore; 9 Post-Acute and Continuing Care, Outram Community Hospital, Singapore, Singapore; 10 Department of Family Medicine and Continuing Care, Singapore General Hospital, Singapore, Singapore; 11 SingHealth Duke-NUS Family Medicine Academic Clinical Program, Singapore, Singapore; 12 SingHealth Office of Regional Health, Changi General Hospital, Singapore, Singapore; University of Coimbra: Universidade de Coimbra, PORTUGAL

## Abstract

**Introduction:**

Many regions in the world are using the population health approach and require a means to measure the health of their population of interest. Population health frameworks provide a theoretical grounding for conceptualization of population health and therefore a logical basis for selection of indicators. The aim of this scoping review was to provide an overview and summary of the characteristics of existing population health frameworks that have been used to conceptualize the measurement of population health.

**Methods:**

We used the Population, Concept and Context (PCC) framework to define eligibility criteria of frameworks. We were interested in frameworks applicable for general populations, that contained components of measurement of health with or without its antecedents and applied at the population level or used a population health approach. Eligible reports of eligible frameworks should include at least domains and subdomains, purpose, or indicators. We searched 5 databases (Pubmed, EMBASE, Web of Science, NYAM Grey Literature Report, and OpenGrey), governmental and organizational sites on Google and websites of selected organizations using keywords from the PCC framework. Characteristics of the frameworks were summarized descriptively and narratively.

**Results:**

Fifty-seven frameworks were included. The majority originated from the US (46%), Europe (23%) and Canada (19%). Apart from 1 framework developed for rural populations and 2 for indigenous populations, the rest were for general urban populations. The numbers of domains, subdomains and indicators were highly variable. Health status and social determinants of health were the most common domains across all frameworks. Different frameworks had different priorities and therefore focus on different domains.

**Conclusion:**

Key domains common across frameworks other than health status were social determinants of health, health behaviours and healthcare system performance. The results in this review serve as a useful resource for governments and healthcare organizations for informing their population health measurement efforts.

## Introduction

Population health has become an increasingly prominent concept in public health discourse, governance, and research in recent years. In their seminal paper, Kindig and Stoddart defines population health as an approach to understanding health that transcends the individual, focusing on interrelated factors and conditions shaping the health of a population. These includes the environment, social and cultural forces, lifestyle choices and government policies [1]. In other words, health cannot be fully understood without a contextualisation of socioeconomic and other factors, such as lifestyle, that are shaped by environments and communities [2]. This change in focus and understanding of health originated during the 1970s-80s in response to the growing body of evidence on social determinants of health, and increasing advocacy for social justice and equity [3]. In contrast to the traditional biomedical model that focused on individual risk factors of diseases, such as obesity, alcohol consumption or family history, a population health approach adopts an upstream preventive approach by addressing root causes, rather than symptoms, to achieve health outcomes.

Population health indicators provide a means for government agencies and Non-Governmental Organisations (NGO) to monitor public health, evaluate interventions, and guide population health policies. Summary measures such as life-expectancy are commonly used to measure the health of a population and for benchmarking against others but are limited on their own, as they do not provide information on other aspects of health [4]. With health and its antecedents being complex and multifaceted constructs, so is the selection of relevant population health indicators. In a scoping review of population health indices, only 7 out of 27 indices had a theoretical or conceptual foundation guiding the aggregation of indicators in a meaningful way [5].

A framework should therefore precede indicator selection [4]. Frameworks provide a structure by which to organise the dynamic and interrelated factors between individuals and their environment, and through which to develop hypotheses about how such relationships affect health outcomes over time [6]. For instance, the widely accepted Canadian Institutes of Health Research population health framework provides an integrated view of health through upstream forces (a whole spectrum of cultural, economic, social and other forces), proximal causes of heath (such as physiological risk factors), lifespan processes, disparities across sub-populations, health services, and health outcomes, as well as the indicators and indices used to measure them [7]. Others may differ depending on their purpose and definition of health and population health.

The usage of a population health framework is necessary as it provides a theoretical grounding and context for selection of indicators and clarifies the role of each indicator [5]. Indeed, this is a step many government agencies and NGOs have taken in their population health efforts. There have been reviews on population health indicators [5, 7, 8]. However, to our knowledge there is no work that organises and clarifies this growing body of literature.

In this paper, we conducted a scoping review with the aim of providing an overview and summary of the characteristics of existing population health frameworks that have been used to conceptualize the measurement of population health. Specific aims were to understand what domains were included in the frameworks, how or why they were chosen, and what some representative indicators under each domain were.

## Methods

This scoping review follows the guidelines described by the Preferred Reporting Items for Systematic reviews and Meta-Analyses extension for Scoping Reviews (PRISMA-ScR) checklist, a minimum set of items for reporting of scoping reviews to promote transparent reporting of scoping reviews [9] (S1 File).

### Eligibility criteria

The eligibility criteria of *population health frameworks* were guided by the elements of the Population, Concept, and Context (PCC) framework. In the population element, we were interested in frameworks that were applied to general populations, which included subsets by demographic variables (e.g. age or ethnicity). However, we excluded populations which were defined by illnesses or diseases (e.g. stroke or mental health patients), or institutional settings (e.g. workplace, schools).

For the Concept element, frameworks should contain components of measurement of health, with or without its antecedents. Frameworks by definition convey structure, at least in the form of categorization [6]. Therefore, eligible frameworks should fulfil this definition. Simple lists of indicators without categories are excluded. Frameworks should also be novel, so mere representations of known literature or frameworks with insufficient explanation, and logic models for specific programs were excluded. For context, frameworks should be applied at the macrolevel, or use a population health approach.

Eligible *reports* of eligible frameworks would need to include at least one of the following dimensions– 1) Domains and subdomains; 2) purpose of the framework; or 3) population health indicators used. Where there were more than 1 report for the same framework, we selected the one with the most relevant and comprehensive information. If another report supplemented information not found in this primary report, we would include both. We included primary articles of any study design, reviews and selected grey literature. Conference abstracts, theses and dissertations, letters to editors, commentaries, non-English articles, and articles published before 1990 were excluded.

### Information sources

We searched MEDLINE (PubMed), EMBASE, Web of Science, NYAM Grey Literature Report and OpenGrey databases. In addition, we searched governmental and organizational sites on Google (site:.gov OR site:.org OR site:.net OR site:.eu) and websites of the following government agencies and NGOs known to have population health initiatives and/or frameworks:

- UK National Health Service (NHS)
- Agency for Healthcare Research and Quality (AHRQ)
- Centres for Disease Control (CDC)
- US Department of Health and Human Services
- Public Health Agency of Canada
- Australian Government Department of Health
- World Health Organization (WHO)
- Organisation for Economic Co-operation and Development (OECD)
- Public Health England
- European Union (EU) CDC
- National Quality Forum (NQF)
- Health Information Technology, Evaluation, and Quality Center (HITEQ)
- The King’s Fund
- Africa Population and Health Research Centre
- Canterbury District Health Board

### Search strategy

We used the keywords ‘framework’ and ‘population health’ from the concept and context elements as search terms, respectively. Depending on the database, we used these terms as keywords or also included controlled vocabulary that corresponded to them. The keywords or controlled vocabulary were combined using the BOOLEAN operator ‘OR’ and ‘AND’ within and across the PCC elements, respectively. The search terms are given in S2 File. Where possible, filters were applied to select only human studies and English articles. The search of the databases was performed from 1 Jan 1990 to 5 May 2023. For some databases (Pubmed, EMBASE, Web of Science) we further applied a ‘title/abstract’ filter to improve the specificity of the search results. If we came across reports that mention an eligible framework but did not contain the relevant details to be included, we then searched for reports on that particular framework. We also searched reference lists of included reports.

### Selection of sources of evidence

Three reviewers (SLC, CZHH, NEEK) developed and piloted the search strategy. Two stages of screenings were performed to select the sources of evidence. At the first stage, the titles and abstracts of each source was screened and selected for full text review by two reviewers independently. In the second stage, the full texts of articles selected in the first stage were also reviewed by 2 reviewers independently. In both stages, a third reviewer would make the final decision in the event of a conflict.

### Data charting process

A data charting form to extract data of interest was developed by one reviewer (SLC) and piloted by another (CZHH). Data from each report was extracted by one reviewer and reviewed by a second reviewer. Any discrepancies were resolved by consensus between the data extractor and reviewer.

### Data items

The data items included citation details, details on the framework (e.g. name, country of origin, organization that developed it, type of population it is applicable to, approach to development, dimensions in framework apart from domains, if framework assessed indicators by certain cross-cutting variables such as life stages, socioeconomic factors, and/or health-related sectors), and the domains and indicators used in the framework, including definitions or descriptions where available. For domains, we recorded up to 2 further levels of sub-domains (total 3 levels).

### Synthesis of results

To facilitate summary and presentation of results, some variables were reduced to a smaller number of categories manually by a single reviewer (SLC). These variables were the type of organization developing the frameworks, types of population the framework was applicable to, and dimensions of the framework. Types of organizations were broadly categorized into governmental, academic, non-government organizations, non-profit organizations, intergovernmental organizations, and private foundations. Populations were grouped in to general, rural and indigenous populations. Finally, dimensions cut across domains and indicators and we focused mainly on a lifespan, health equity and sector approach. For the lifespan approach, this generally involve diving into indicators relevant for different life stages and/or breaking down indicators by age groups. For the equity approach this typically involves examining indicators by certain socioeconomic factors, such as education level, income, and ethnicity. For the sector approach, this involves looking at indicators specific for different health-related sectors such as clinical care, public health, and community and social services. We categorized frameworks under ‘dimensions’ into lifespan, equity and/or other specific dimensions mentioned.

The characteristics of the frameworks were then summarized descriptively using counts and proportions, and median and ranges, as appropriate. Domains were aggregated by concept using hierarchical clustering and manual refinement for purposes of visualization. The final clustering was agreed on by 3 reviewers (SLC, CP, JSG). The domain concepts, and number of domains, subdomains and indicators were visualized using a word cloud and heatmap, respectively. Other aspects of the frameworks were summarized narratively.

## Results

### Search results

**Fig 1:**
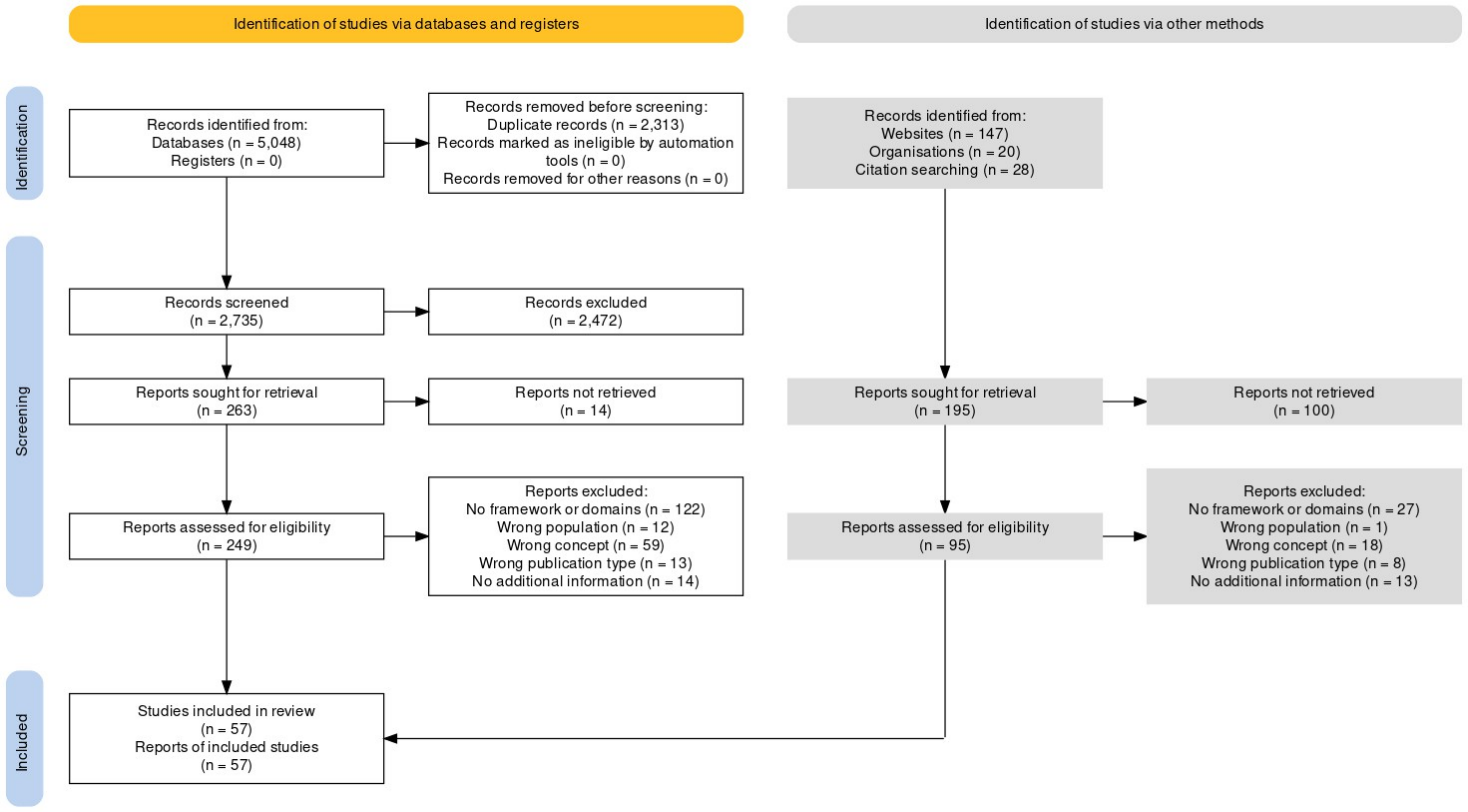
PRISMA diagram. The PRISMA diagram shows the numbers of reports retrieved from various sources and flow through the stages of the scoping review. A total of 57 reports were included in this review. The diagram was generated using an open source R shiny app [10].

A total of 57 population health frameworks were included in this review (Fig 1). The characteristics of the frameworks and their details are shown in Tables 1 and 2, respectively. The full list of the domains, subdomains and indicators are provided in S3 File.

**Table 1:** Summary of key characteristics of included frameworks.

Characteristics	N (%)*
Year of publication	
2000 and before	6 (10.5)
2001 to 2010	15 (26.3)
2011 to 2020	22 (38.6)
2020 onwards	14 (24.6)
Country/region of origin	
US	26 (45.6)
Europe	13 (22.8)
Canada	11 (19.3)
Australia/New Zealand	3 (5.3)
International	2 (3.5)
US and Western Europe	1 (1.8)
Ghana	1 (1.8)
Type of organization framework originated from	
Governmental	23 (40.4)
Academic	16 (28.1)
Non-profit organization	8 (14.0)
Intergovernmental	4 (7.0)
Governmental/academic	3 (5.3)
Governmental/non-profit organisation	1 (1.8)
Intergovernmental/academic/non-governmental organisation	1 (1.8)
Private foundation	1 (1.8)
Population framework is applied to	
General/urban	54 (94.7)
Indigenous	2 (3.5)
Rural	1 (1.8)
Dimensions	
None	19 (33.3)
Lifespan	17 (29.8)
Equity	12 (21.1)
Lifespan and equity	8 (14.0)
Sector	1 (1.8)

*Percentages may not add up to 100% due to rounding

**Table 2:** Details of included frameworks.

Ref / Year of publication	Framework name	Country/region of origin	Name of organization that developed it (Type of organization)	Population	Dimensions^†^
Arah 2005 [11]	Canadian Health Indicators Framework (modified)	Canada	Canadian Government (Gov)	General/Urban	Equity
Azzopardi 2018 [12]	Reporting framework for Indigenous adolescents in Australia	Australia	University of Melbourne, Murdoch children’s research institute (Acad)	Indigenous	
Beard 2009 [13]	Framework for considering the influence of socioeconomic and cultural factors on health	Australia	Northern Rivers University Department of Rural Health (Acad)	Rural	
Casebeer 1999 [14]	Health indicators framework	Canada	Collaborative initiative of the Alberta Heritage foundation for medical research and Alberta Health (Acad)	General/Urban	
CDC 2013 [15]	A Schematic Framework for Population Health Planning	US	U.S. Department of Health and Human Services & Centres for Disease Control and Prevention (Gov)	General/Urban	
CHS 2021 [16]	System Level Measures (SLMs) Framework	New Zealand	Canterbury Health System	General/Urban	Lifespan, equity
CIHI 2013 [17]	Canadian Institute for Health Information (CIHI)’s New Health System Performance Measurement Framework	Canada	CIHI (NPO)	General/Urban	Equity
Emeny 2022 [18]	Precision Health Framework	US	University of New Mexico	General/Urban	
Etches 2006 [7]	Canadian Institutes of Health Research (CIHR)—Institute of Population and Public Health (IPPH) conceptual framework of population health	Canada	CIHR and IPPH (Gov)	General/Urban	
EU 2015 [19]	Joint Assessment Framework	Europe	European Union (EU)/European Commission (Inter-Gov)	General/Urban	Lifespan, equity
Evans 1990 [20]	Evans and Stoddart	Canada	University of British Columbia, McMaster University (Acad)	General/Urban	
Galea 2005 [21]	Conceptual framework for urban health	US	Center for Urban Epidemiologic Studies, New York Academy of Medicine (Acad)	General/Urban	Equity
Halfon 2002 [22]	Life course health development framework	US	National centre for Infancy and early childhood health policy (Gov)	General/Urban	Lifespan
Hancock 1999 [23]	Basic Framework for indicators	Canada	Knowledge Development Division, Health Canada (Gov)	General/Urban	Equity
Hatef 2018 [24]	Maryland	US	Maryland Department of Health (Gov)	General/Urban	Lifespan
Health Canada 1994 [25]	Framework for action on population health	Canada	Federal/provincial/territorial advisory committee on population health (Gov)	General/Urban	
Health Policy Institute of Ohio 2016 [26]	Ohio Health Priorities	US	Ohio Governor’s Office of Health Transformation, Ohio Department of Health and Ohio Department of Medicaid	General/Urban	Lifespan
Healthy Montgomery 2016 [27]	Healthy Montgomery Core Measures Set	US	Montgomery County Department of Health and Human Services (Gov)	General/Urban	
Healthy Ireland 2019 [28]	Healthy Ireland (HI) Outcomes Framework	Ireland	Ireland Department of Health (Gov)	General/Urban	Lifespan
Hillemeier 2003 [29]	Framework for community contextual characteristics	US	The authors in collaboration with the CDC (Gov)	General/Urban	
Hood 2016 [30]	County Health Rankings	US	University of Wisconsin Population Health Institute and the Robert Wood Johnson Foundation (Acad)	General/Urban	Lifespan, equity
Inf-Act 2020 [31]	A Distributed Infrastructure on Population Health (DiPoH)	Europe	Information for Action (InfAct) (Gov)	General/Urban	
IOM 2009 [32]	Institute of Medicine (IOM)	US	National Academy of Medicine (formerly IOM until 2015) (NPO)	General/Urban	Lifespan
IOM 2012 [33]	Healthy People 2020 Leading Health Indicators (Health Outcome Logic Model)	US	National Academy of Medicine (formerly IOM until 2015) (NPO)	General/Urban	Lifespan
IP3 2017 [34]	Vital Conditions Framework	US	Institute for People, Place, & Possibility	General/Urban	Lifespan
Ireland Department of Health 2021 [34]	Health System Performance Assessment (HSPA) Framework	UK	Ireland Department of Health	General/Urban	Lifespan/equity
Jeffery 2006 [35]	Box framework for population health indicators	Canada	The authors in collaboration with the Inuit Tapiriit Kanatami, Prince Albert Grand Council (PAGC) and Athabasca Health Authority (AHA) (Gov/(NPO)	Indigenous	
Juarez 2014 [36]	Public Health Exposome Conceptual Model	US	University of Tennessee Health Science Center (Acad)	General/Urban	Lifespan, equity
Kassler 2017 [37]	Centers for Medicare & Medicaid Services (CMS)	US	CMS (Gov)	General/Urban	Sector (clinical care, public health, community & social services)
Kim 2013 [38]	Social Determinants of Infant Mortality/Birth Outcomes Conceptual Framework	US and Western Europe	RAND corporation (NPO)	General/Urban	Equity
Kramers 2003 [39]	European Community Health Indicators (ECHI)	Europe	European Commission (Gov)	General/Urban	Lifespan, equity
Krewski 2007 [40]	An integrated framework for risk management and population health	Canada	University of Ottawa (Acad)	General/Urban	
Kuehnert 2021 [41]	Not reported	US	American Academy of Nursing	General/Urban	Lifespan
Kumah 2020 [42]	Ghana’s Holistic Assessment Tool	Ghana	Ghana’s Ministry of Health (Gov)	General/Urban	Lifespan, equity
LA County 2017 [43]	Los Angeles (LA) Key indicators of health	US	LA County Department of Public Health (Gov)	General/Urban	Lifespan
Levene 2018 [44]	Leicester Systematic Exploration and Analysis of Relation-ships Connecting Health variables in populations (SEARCH)	UK	George Davies Centre for Medicine	General/Urban	
NQF 2014 [45]	National Quality Forum (NQF) population health indicators	US	NQF (NPO)	General/Urban	Lifespan
OECD 2021 [46]	Organisation for Economic Co-operation and Development (OECD) Framework for health system performance assessment	International	OECD (Inter-Gov)	General/Urban	Lifespan
Oleske 2009 [47]	Oleske epidemiologic model for the delivery of health care services	US	*Not reported*	General/Urban	
PHCPI 2022* [48]	Primary Healthcare Performance Initiative (PHCPI) conceptual framework	International	World Health Organization (WHO), World Bank Group, and the Bill & Melinda Gates Foundation, in partnership with Ariadne Labs and Results for Development Institute (Inter-Gov/NGO/Acad)	General/Urban	Equity
PHE 2021 [49]	Labonte model	UK	Public Health England (PHE) (Gov)	General/Urban	Equity
Robine 2002 [50]	Euro-REVES 2	Europe	Euro-REVES group (Acad)	General/Urban	
Roos 1995 [51]	Population Health Information System	Canada	Manitoba Centre for Health Policy and Evaluation (Acad)	General/Urban	Lifespan
Sadana 2002 [52]	WHO Multi-Country Survey	Switzerland	WHO (Inter-Gov)	General/Urban	Lifespan
Santana 2020 [53]	EURO-HEALTHY Population Health Index model	Europe	Centre of Studies in Geography and Territorial Planning (Acad)	General/Urban	Equity
Schoen 2006 [54]	National scorecard for the US health system	US	Commonwealth Fund Private foundation	General/Urban	Equity
Schoon 2022 [55]	Holistic Health Determinants Model	US	Minnesota State University Mankato	General/Urban	Lifespan/equity
Schulz 2004 [56]	Social Determinants of Health and Environmental Health Promotion	US	School of Public Health, University of Michigan (Acad)	General/Urban	Equity
SDH 2020 [57]	Live Well San Diego equity framework	US	County of San Diego Health and Human Services Agency	General/Urban	
SfHIP 2022* [58]	San Francisco Framework for assessing population health and equity	US	San Francisco Health Improvement Partnership (SfHIP) (Gov/Acad)	General/Urban	Equity
Shah 2017 [59]	Health Equity Framework	US	Harris County Public Health, Texas (Gov)	General/Urban	Equity
Stiefel 2012 [60]	Triple Aim	US	Institute for Healthcare Improvement (NPO)	General/Urban	Lifespan
ten Asbroek 2004 [61]	Dutch performance indicator framework	Netherlands	Department of Social Medicine, Academic Medical Centre, University of Amsterdam; Dutch Ministry of Health, Welfare, and Sports (Gov/Acad)	General/Urban	
UK Department of Health 2022* [62]	Public Health Outcomes Framework	UK	Department of Health (Gov)	General/Urban	Lifespan
Vila 2006 [63]	Wisconsin County Health Rankings	US	University of Wisconsin Population Health Institute (Gov/Acad)	General/Urban	Lifespan
Webster 2013 [64]	Healthy Cities Indicators	Europe	WHO European Healthy Cities Network (Inter-Gov)	General/Urban	
Wolfson 1994 [65]	Population Health Model (POHEM)	Canada	Statistics Canada (Gov)	General/Urban	Lifespan

*These are websites and the year is based on the date of access,

^†^not all frameworks explicitly mentioned a dimension.

Acad: academic, Gov: government, Inter-gov: inter-government, NGO: non-government organisation, NPO: non-profit organisation

### Characteristics of population health frameworks

Majority of the frameworks originated from the US (45.6%), Europe (22.8%) and Canada (19.3%). None were from Asia. Most were published between 2001 and 2020 (64.9%). Governmental (including intergovernmental) and academic organizations accounted for majority of framework development (84.2%). Only three frameworks were developed for specific populations (2 for indigenous and 1 for rural), while the rest were for the general or urban population. Two-thirds of the frameworks mentioned some dimension, and these were slightly more frameworks using the lifespan approach compared to the equity approach (29.8% vs. 21.1%).

### Domains and subdomains

Majority of the frameworks have between 1 to 5 domains (70.2%) but have more level 2 sub-domains (26.3% have 6–10, 29.8% have 11–20 and 19.3% have >20). The median number of domains and level 2 subdomains are 4 (range 2–16) and 10 (range 0–65), respectively (S1 Fig). Half of the frameworks do not have level 3 subdomains. Of those that do, most have >10 (72.4%). The median number of indicators is 18 (range 0–255). Twenty-six frameworks did not have indicators (45.6%). Of those that do, majority have >20 indicators (83.9%).

The most common concepts were health, (social) determinants of health, healthcare system and health behaviours (S2 Fig). The myriad of domains has gradually accumulated over the years. In frameworks published before 2000, health was the key domain, social determinants of health emerged in the next 2 decades (2001–2020) followed by healthcare system, health behaviours, functional limitations and activities of daily living in the recent frameworks (S3 Fig).

For health, most frameworks used summary indicators of health such as mortality and life-expectancy, and indicators of a few selected health conditions. However, four frameworks had longer lists of indicators for specific communicable and non-communicable diseases [12, 26, 27, 43, 50]. Of note, psychological or mental health risk factors and/or outcomes feature in 31 (54%) of the frameworks, highlighting its emerging importance [12, 17–19, 22, 25–30, 32–35, 38, 39, 41–46, 48–50, 54, 56, 58, 59, 62].

Social determinants of health, which encompasses the full set of social conditions in which people live and work [66], were present under some label or other in all except 7 frameworks [16, 34, 42, 50, 52, 54, 60]. Some of the frameworks elaborate on these factors, with sub-domains and indicators on the physical environment, social environment, and even politics, national and global trends [12, 21–23, 26, 29, 35, 43, 53, 55–59, 63, 64, 67]. For example, the conceptual framework for urban health measures sub-domains such as immigration, globalization and the changing role of government [21]. The framework for community contextual characteristics, one of the two frameworks with the largest number of indicators, also measures the economic, employment, education, political, environmental, housing, governmental, transport aspects in the region where the population of interest is located [29]. Interestingly, crime and violence features in 16 frameworks, as this affects the physical safety of people in a community [12, 15, 26, 29, 30, 33, 41, 43–45, 53, 56, 59, 62, 63, 67]. Many frameworks also measure lifestyle and health-related behaviours. Apart from the common ones like diet, physical activity, smoking and alcohol use, some frameworks include sexual behaviour, use of illicit drugs, seatbelt behaviour, immunization or health screening, breastfeeding and induced abortion [12, 15, 27–30, 32, 33, 39, 45, 55, 58, 59, 62]. One even included measures of parenting practices [43].

Almost a third (31.6%) of the frameworks have domains that pertain to the healthcare system or healthcare performance. One example is the OECD framework, which assesses health system performance within the context of other contextual determinants of health [46]. Within the construct of healthcare performance, common subdomains are accessibility, capacity, quality, patient-centeredness, cost and effectiveness [11, 16, 19, 24, 31, 32, 34, 39, 43, 46, 48, 51, 53, 54].

A few of the frameworks had specific focuses and therefore unique domains and indicators that are relevant largely for their setting. For example, the reporting framework for indigenous adolescents in Australia contained domains that were largely relevant for that community, such as ‘family, kinship and community health’, which explored family roles and responsibilities, contact with extended family, removal from family, participation in community events and sense of belonging to the community [12]. Another example is the Ghana’s Holistic Assessment Tool, which contains indicators for health-related United Nations sustainable development goals (SDGs) such as proportion of deliveries attended by a trained health worker, proportion of children under 5 years sleeping under insecticide treated net, and tuberculosis treatment success rate, and certain endemic communicable diseases such as non-acute flaccid paralysis polio rate [42].

### Approach to framework development

Evans and Stoddart developed a population health framework in 1990 [20] based on a much earlier 1974 Whitepaper titled “A new perspective on the health of Canadians”, which recognized the limitations of the healthcare system on improving health status and presented a preliminary framework of the ‘health field’ [68]. Subsequent frameworks were mostly developed from one or a combination of four approaches: 1) adaptation from an existing framework [11, 12, 33, 45, 46, 48–51, 56, 58–60, 63, 65], 2) environmental scan of existing frameworks and literature review to summarize current knowledge of health determinants [7, 14, 16–20, 24, 25, 29, 32, 36, 37, 44, 48, 52, 57, 61, 63], 3) consulting and getting inputs from experts and stakeholders [12, 17, 19, 24, 26–29, 35, 39, 41, 48, 52–55, 62, 63] and 4) basing on past work (e.g. primary data collection, drawing on secondary data, past population health efforts, etc), priorities and goals of the organization developing it [7, 11, 21, 38, 61, 64, 67].

## Discussion

Population health has been a popular concept in healthcare for the past 3 decades but interestingly does not have a unanimous definition [1, 2, 69]. The most commonly used definition, which originated from Kindig and Stoddart, defines population health as ‘the health outcomes of a group of individuals, including the distribution of such outcomes within the group” [1]. Nevertheless, people working on ‘population health’ would have different focuses, goals and populations of interest [69]. This may explain the large number of population health frameworks we found in this review.

Population health has its roots from recognition of health disparities by socioeconomic factors from as early as the 18^th^ century to early epidemiological studies that informed public health measures, particularly in Britain and France, and finally to a renewed interest in the last 2 decades due to a range of health problems facing the world [70]. Development of the population health approach in Canada, driven by the government and healthcare leaders, began in the 1970s [71]. Improving population health was motivated by the articulation of the Triple Aims as a goal for the US healthcare system in the late 2000s [72]. It is therefore unsurprising that most of the frameworks originate from US, Europe and Canada. Even with purposive searching of organizations in the Southern hemisphere such as Australia and New Zealand, the results were still dominated by the Northern hemisphere, reflecting the state of development of population health in the world. Similarly, the lack of frameworks from Asia might be because much of the work done in improving the health of populations is ‘public health’ rather than ‘population health’.

Health status and social determinants of health were the most common domains across the frameworks. As seen from the word cloud, there were also many other domains that were closely related to and/or could be considered subdomains of one of these domains. This is because different frameworks have different level of detail, and the hierarchy of domains and subdomains are different in level of detail across frameworks. In other words, a subdomain in one framework could be a domain in another, or an indicator in one framework could be a subdomain in another. It is therefore also difficult to summarize domains and subdomains in a simple way across the frameworks.

The domains and subdomains chosen in different frameworks largely reflects the purpose, information needs of varying stakeholders, and the focus of the organization(s) developing them. It is unsurprising to see that some key domains appear in many frameworks, and domains are branched out to varying degrees in different frameworks. For example, social determinants of health features in all frameworks except 7 frameworks [16, 34, 42, 50, 52, 54, 60]. Some frameworks have a heavy focus on health status, such as the Healthy Montogomery Core Measures Set, Triple Aim, Euro-REVES 2 and Ohio health priorities, with the Euro-REVES 2 framework even measuring activities of daily living and degree of functional limitations [26, 27, 50, 60]. Other frameworks break down the social determinants into considerable detail, such as the framework for community contextual characteristics, life course health development framework, Healthy Cities Indicators, and others [12, 22, 23, 26, 29, 38, 49, 53, 55, 56, 59, 63, 64, 67]. Several have a heavier focus on healthcare performance, such as the EU Joint Assessment Framework, European Community Health Indicators (ECHI), OECD, the Primary Healthcare Performance Initiative (PHCPI), National scorecard for the US health system and the Ireland HSPA framework [19, 34, 39, 46, 48, 54]. Others are generally more balanced between the domains.

It is also noteworthy that almost half of the frameworks did not have any indicators and these tended to be older frameworks. About 61% of frameworks developed in 2010 and before did not have indicators while the converse is true for those developed after 2010. There was likely stronger focus on understanding the range of factors affecting population health and identifying priorities for improving population health in the earlier period. As organizations started to implement population health management strategies, measurement of population health started to feature more and more recent frameworks tended to include specific indicators. The inclusion of specific indicators also implies the ability to measure them, and therefore the availability of health information systems for data collection. These have generally become more well developed in the recent decade or so, also explaining why more recent frameworks have indicators. Nevertheless, frameworks without indicators can still offer a theoretical basis for selecting indicators that are relevant and feasible for a given setting.

The results of this scoping review can serve as an evidence base for governments and/or health systems developing their own population health frameworks and selecting indicators for their population health initiatives. They can select and adapt from the frameworks available, and assess the relevance of the range of domains, subdomains and indicators in their context. Populations are largely unique as they are shaped by their local and wider contextual factors. As such, no one framework used in one population or healthcare system is likely directly applicable to another population or healthcare system without adaptation. Population health practitioners can derive any level of detail that matches their interests and requirements from this review, from a broad sense of the literature down to specific indicators. The range of subdomains and indicators could also be sources of new hypotheses in a given region or jurisdiction for the purposes of population health research.

Settings which are further ahead in the population health journey with existing indicators can also use these results to assess what domains and subdomains have been covered, and where the gaps are. For example, population health is an increasingly important national priority in Singapore and the Ministry of Health is planning several major initiatives to improve the health of the general population [73, 74]. To achieve this, the Ministry is working closely with the three major public healthcare clusters in Singapore to develop a set of population health indicators and the evidence base here can help inform the choices. With an initial set of indicators, practitioners can also interrogate their data systems and medical records to determine if they are available or if they need to build prospective data collection tools. This can also be an iterative process for selecting indicators using the results here as a resource. One constraint of the data in its current form though is the difficulty in navigating the long list of domains, subdomains and indicators. In future work, we aim to design a dashboard that allows for interactive exploration of the scoping review data.

There are limitations to this scoping review. Firstly, some frameworks might have been missed due to our language restriction, especially those in Asia. However, many official documents from this region are available in English, so this might not have impacted the search results significantly. Secondly, there are many terms and concepts in the literature that have overlaps with population health, such as public health, urban health, global health, population health management, health equity, health system performance and social determinants of health. Based on our inclusion criteria, concepts like urban health, rural health, community health and global health would be included as they pertain to general populations albeit in different types of settings. Related concepts such as health equity, social determinants of health and health system performance were not the focus of the search and could be part of the frameworks included. However, if a framework was focused on one of these concepts alone without the measurement of health status, then it would be excluded. Some frameworks also focused more on population health management and if it looked more like a logic model for specific interventions then these would also be excluded [75, 76]. Overall, this review represents a useful collection of frameworks used for measuring the health of a population and its key antecedents [60].

## Conclusion

We found 57 frameworks for the measurement of population health with variable numbers of domains, subdomains and indicators, and depth of detail. The key domains apart from health status were social determinants of health, health behaviours and healthcare system performance. These results serve as a useful resource for governments and healthcare organizations for informing their population health measurement efforts. Specifically, when developing their own population health framework and/or selection of population health indicators, they can identify common domains and subdomains that other organizations include, as well as consider others more systematically for relevance in their context.

## Supporting information

S1 File PRISMA-ScR checklist. (DOCX)

S2 File Search strategy. (DOCX)

S3 File Domains, subdomains and indicators. This file contains the full list of domains, subdomains and indicators from the 57 included population health frameworks.
(XLSX)

S1 Fig Heatmap of number of domains, subdomains and indicators. L2: level 2, L3: level 3, This is a visualization of the numbers of domains, subdomains and indicators in each framework in both figures and shading. Blank cells represent absence of the corresponding subdomain and/or indicators.
(DOCX)

S2 Fig Wordcloud for framework domains. Level 1 domains in all frameworks were clustered by concept using a combination of hierarchical clustering and manual edit. The sizes of the concepts are proportional to the number of domains in each concept.
(DOCX)

S3 Fig Wordcloud for framework domains by year of publication. Level 1 domains in all frameworks were clustered by concept using a combination of hierarchical clustering and manual edit. The sizes of the concepts are proportional to the number of domains in each concept. The concepts are presented by decade when the frameworks were published. A: Before 2000, B: 2001 to 2010, C: 2011 to 2020, D: After 2020.
(DOCX)
