# Supplementary material for: Frameworks for measuring population health: A scoping review
Source: PLoS One. 2024 Feb 13;19(2):e0278434. doi: 10.1371/journal.pone.0278434 (PMC10863900; doi:10.1371/journal.pone.0278434)
Supplement: S2 File — (DOCX) [file pone.0278434.s002.docx]

**Supplementary file 2 - Search strategy**

Keywords and controlled vocabulary

| **Concept** | **Keywords** | **Pubmed (Mesh terms)** | **Embase (Emtree terms)** |
| --- | --- | --- | --- |
| Concept - measurement frameworks | framework |  |  |
| Context | population | population health | population health |
|  | population health |  |  |

Queries

| **Database** | **Query** |
| --- | --- |
| Pubmed | (("population health"[Mesh]) OR ("population health"[tiab])) AND (framework*) Filters: English, Humans, from 1990/1/1-2023/5/5 |
| Embase | ('population health'/exp OR 'population health':ti,ab) AND framework* AND [humans]/lim AND [english]/lim AND [1990-2022]/py |
| Web of Science | ((TI=("population health")) OR AB=("population health")) AND (ALL=(framework*)) and English(Languages) |
| NYAM Grey Literature Report | "population health" framework |
| OpenGrey | "population health" AND framework* |
| Google | "population health" framework* (site:.gov OR site:.org OR site:.net) |
| Website search - NHS UK | england.nhs.uk |
| Website search - AHRQ | https://www.ahrq.gov/ |
| Website search - CDC | https://www.cdc.gov/ |
| Website search - US dept of HHS | https://www.hhs.gov/ |
| Website search - Public Health Agency of Canada | [https://www.canada.ca/en/public-health.html](about:blank) |
| Website search - Australian Government Dept of Health | https://www.health.gov.au/ |
| Website search - WHO | https://www.who.int/ |
| Website search - OECD | https://www.oecd.org/ |
| Website search - Public Health England | https://www.gov.uk/government/organisations/office-for-health-improvement-and-disparities |
| Website search - EU CDC | https://www.ecdc.europa.eu/en |
| Website search - NQF | [https://www.qualityforum.org/Home.aspx](about:blank) |
| Website search - EU Public Health | https://ec.europa.eu/health/index_en |
| Google | "population health" framework* (site:.eu) |
| Website search - HITEQ | [https://hiteqcenter.org/](about:blank) |
| Website search – The King’s Fund | https://www.kingsfund.org.uk/ |
| Website search – Africa Population and Health Research Centre | https://aphrc.org/ |
| Website search – Canterbury District Health Board | https://www.cdhb.health.nz |
