# Supplementary material for: Frameworks for measuring population health: A scoping review
Source: PLoS One. 2024 Feb 13;19(2):e0278434. doi: 10.1371/journal.pone.0278434 (PMC10863900; doi:10.1371/journal.pone.0278434)
Supplement: S1 Fig — L2: level 2, L3: level 3, This is a visualization of the numbers of domains, subdomains and indicators in each framework in both figures and shading. Blank cells represent absence of the corresponding subdomain and/or indicators. (DOCX) [file pone.0278434.s004.docx]

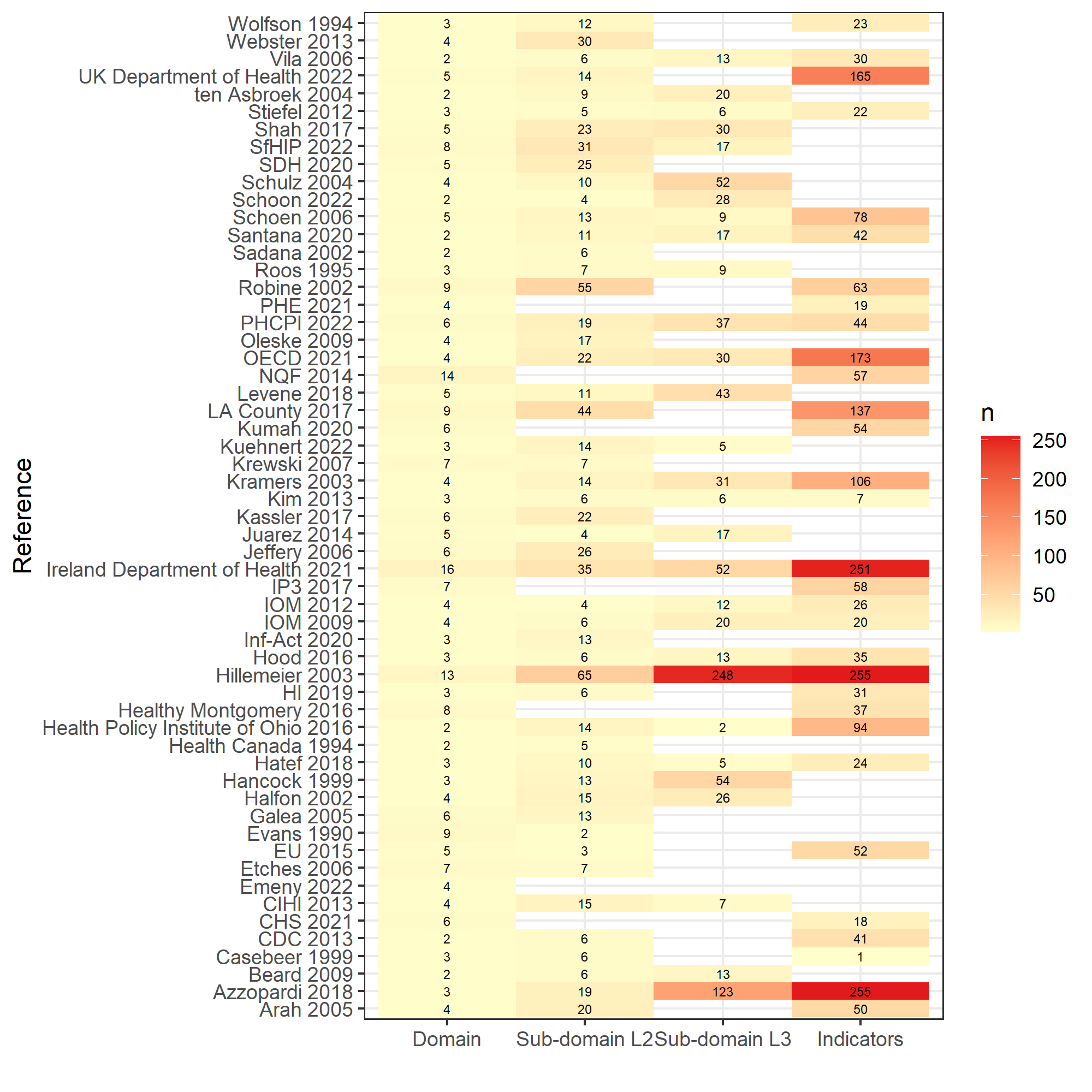


Supplementary Figure 1 Heatmap of number of domains, subdomains and indicators

L2: level 2, L3: level 3

This is a visualization of the numbers of domains, subdomains and indicators in each framework in both figures and shading. Blank cells represent absence of the corresponding subdomain and/or indicators.
