# Supplementary material for: Frameworks for measuring population health: A scoping review
Source: PLoS One. 2024 Feb 13;19(2):e0278434. doi: 10.1371/journal.pone.0278434 (PMC10863900; doi:10.1371/journal.pone.0278434)
Supplement: S2 Fig — Level 1 domains in all frameworks were clustered by concept using a combination of hierarchical clustering and manual edit. The sizes of the concepts are proportional to the number of domains in each concept. (DOCX) [file pone.0278434.s005.docx]

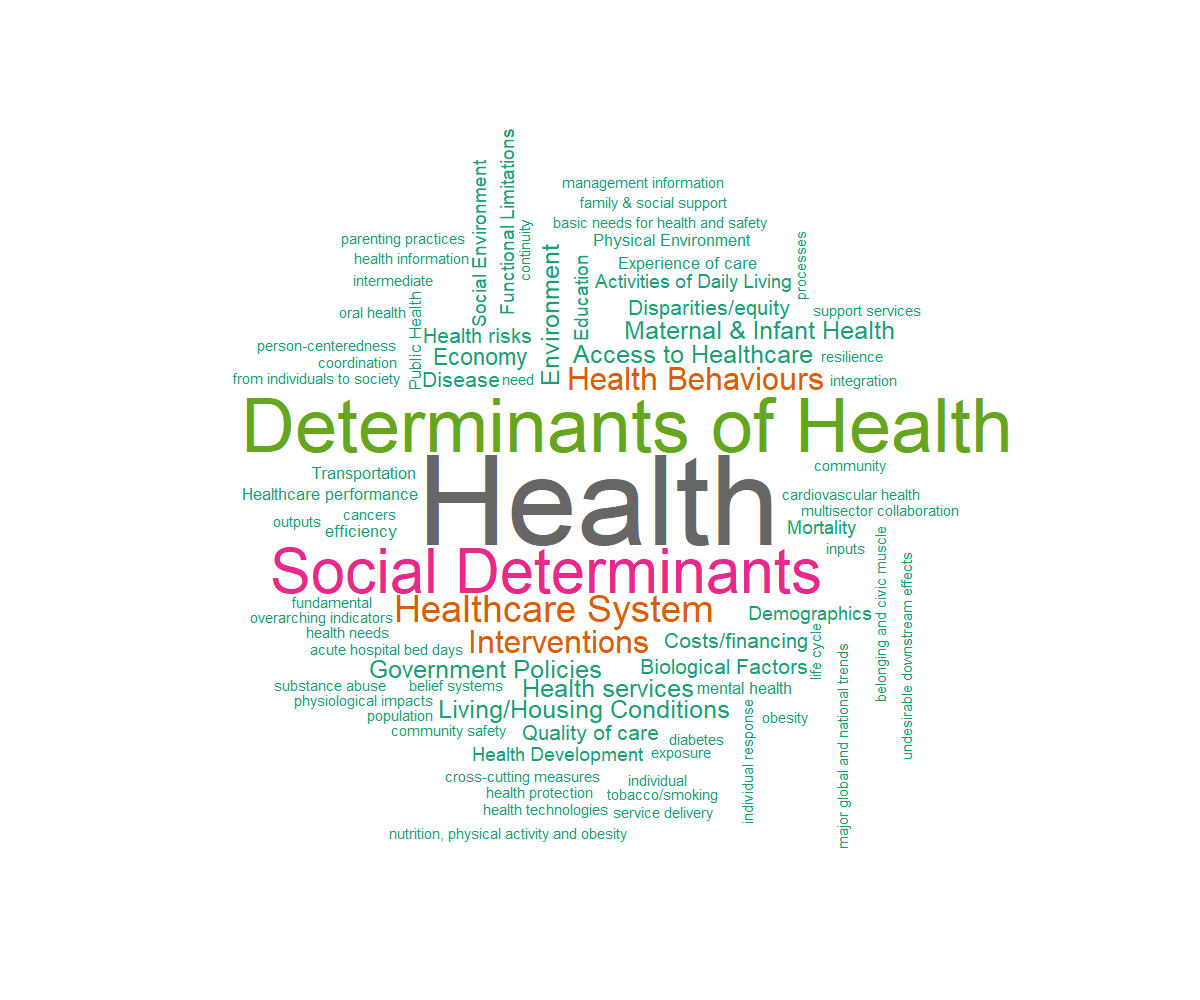


Supplementary Figure 2 Wordcloud for framework domains

Level 1 domains in all frameworks were clustered by concept using a combination of hierarchical clustering and manual edit. The sizes of the concepts are proportional to the number of domains in each concept.
