# Supplementary material for: Frameworks for measuring population health: A scoping review
Source: PLoS One. 2024 Feb 13;19(2):e0278434. doi: 10.1371/journal.pone.0278434 (PMC10863900; doi:10.1371/journal.pone.0278434)
Supplement: S3 Fig — Level 1 domains in all frameworks were clustered by concept using a combination of hierarchical clustering and manual edit. The sizes of the concepts are proportional to the number of domains in each concept. The concepts are presented by decade when the frameworks were published. A: Before 2000, B: 2001 to 2010, C: 2011 to 2020, D: After 2020. (DOCX) [file pone.0278434.s006.docx]

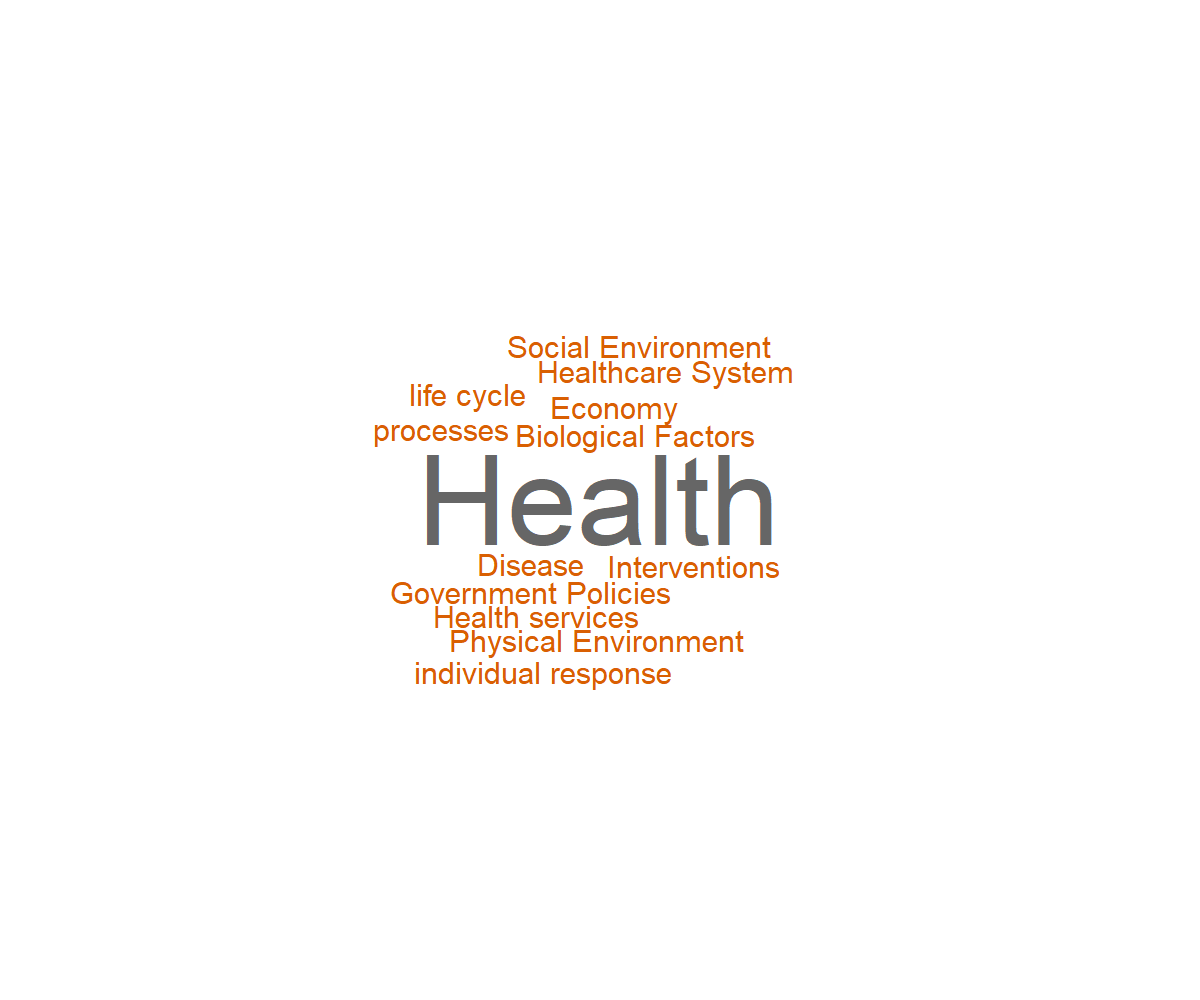


**A**


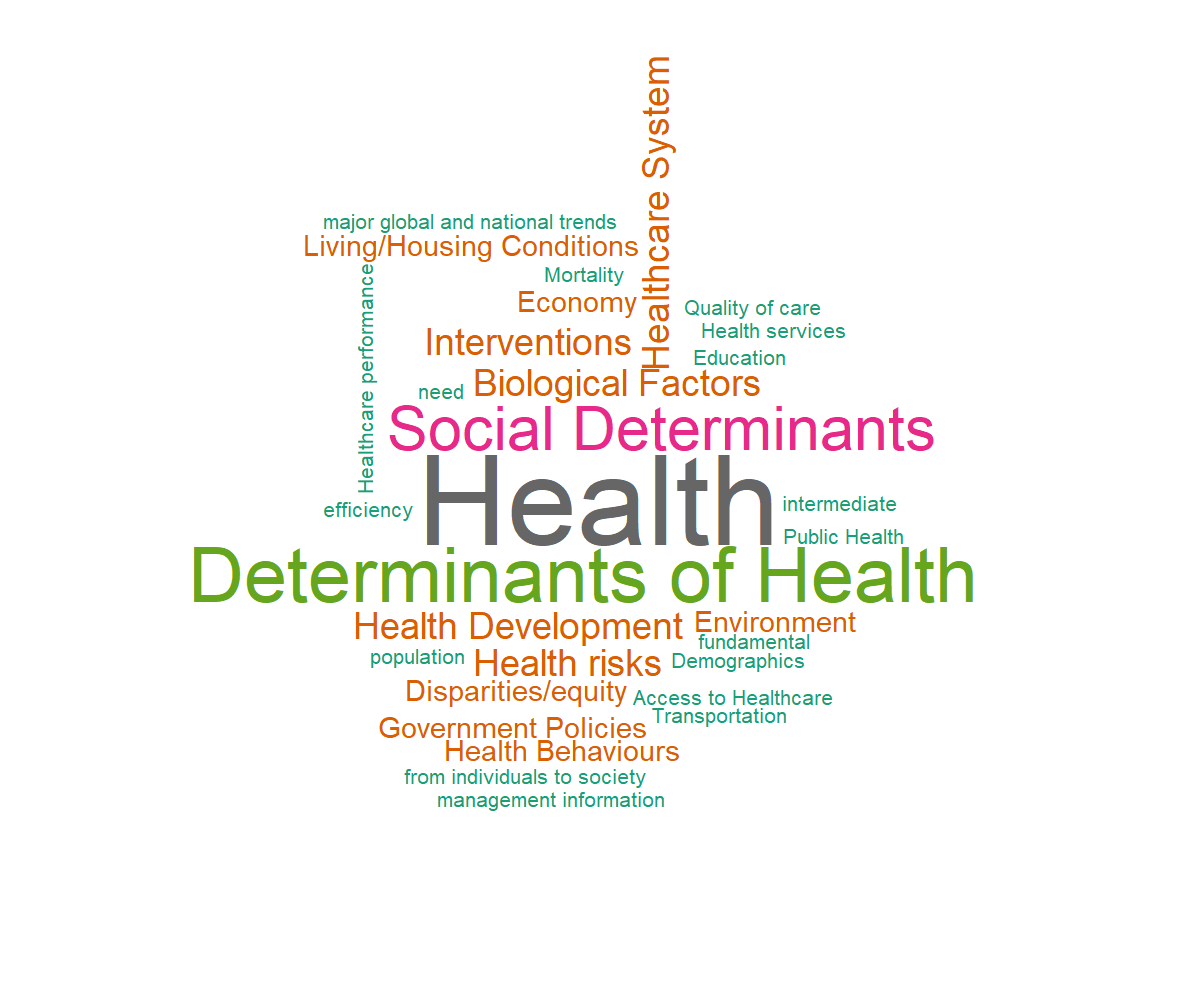


**B**


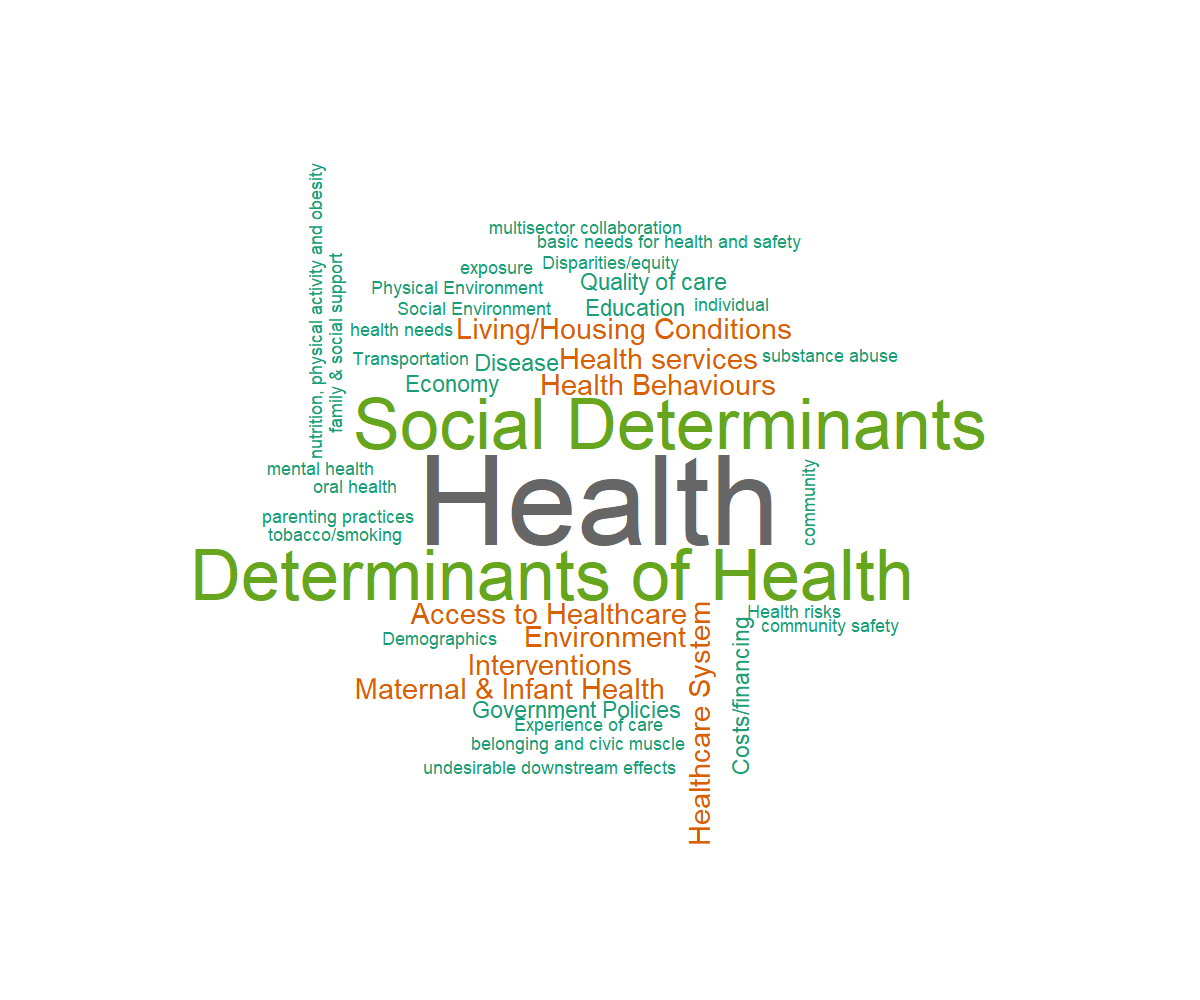


**C**


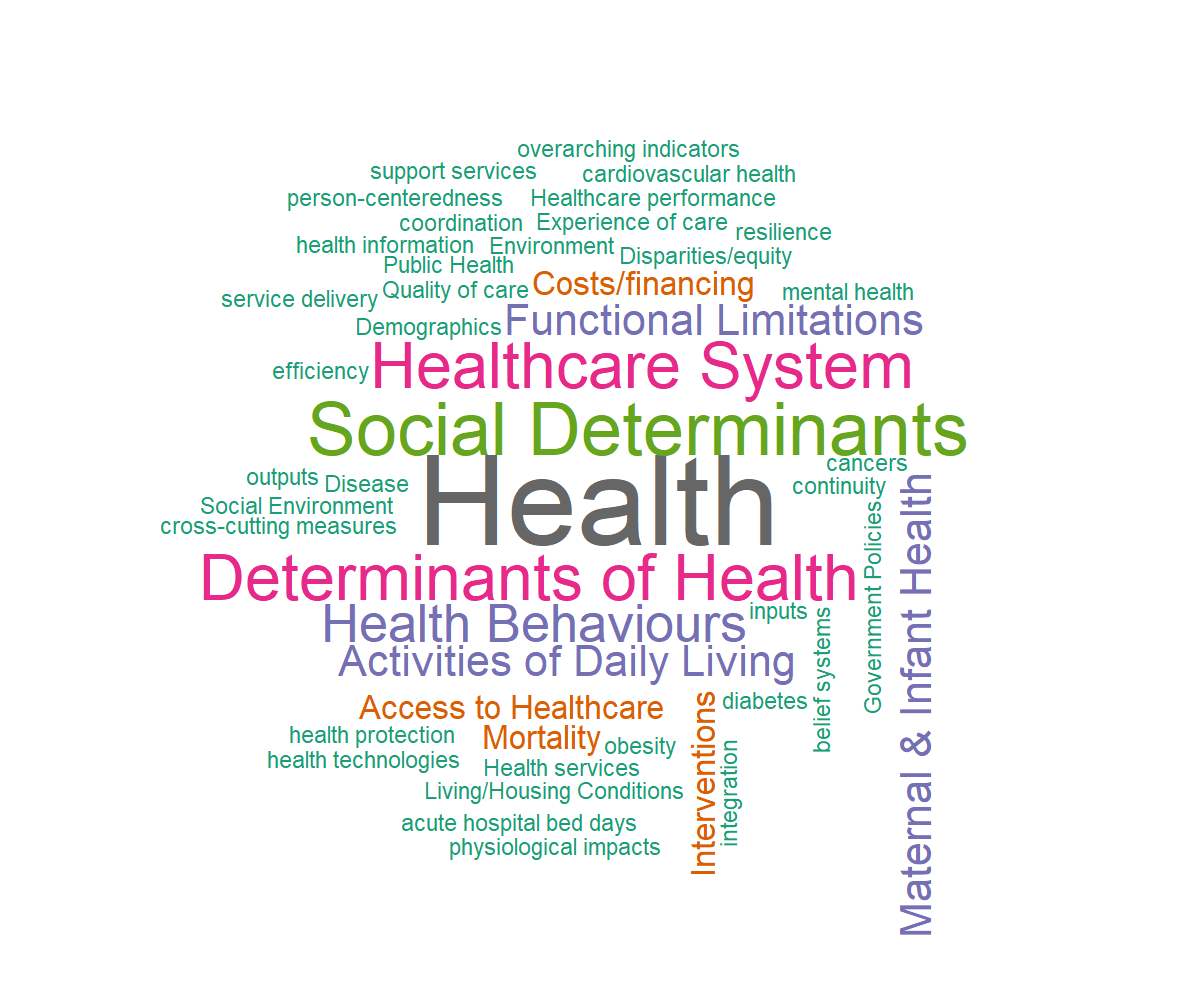


**D**

Supplementary Figure 3 Wordcloud for framework domains by year of publication

Level 1 domains in all frameworks were clustered by concept using a combination of hierarchical clustering and manual edit. The sizes of the concepts are proportional to the number of domains in each concept. The concepts are presented by decade when the frameworks were published. A: Before 2000, B: 2001 to 2010, C: 2011 to 2020, D: After 2020
